# A New Insight into the Role of CART in Cocaine Reward: Involvement of CaMKII and Inhibitory G-Protein Coupled Receptor Signaling

**DOI:** 10.3389/fncel.2017.00244

**Published:** 2017-08-15

**Authors:** ChengPeng Yu, XiaoYan Zhou, Qiang Fu, QingHua Peng, Ki-Wan Oh, ZhenZhen Hu

**Affiliations:** ^1^The Second Clinic Medical College, School of Medicine, Nanchang University Nanchang, China; ^2^Department of Pathophysiology, College of Medicine, Nanchang University Nanchang, China; ^3^Department of Respiration, The Fourth Affiliated Hospital, Nanchang University Nanchang, China; ^4^Department of Respiration, Department Two, Jiangxi Provincial People’s Hospital Nanchang, China; ^5^Department of Anesthesiology, The First Affiliated Hospital, Nanchang University Nanchang, China; ^6^College of Pharmacy, Chungbuk National University Cheongju, South Korea; ^7^Jiangxi Province Key Laboratory of Tumor Pathogens and Molecular Pathology and Department of Pathology, Schools of Basic Medical Sciences and Pharmaceutical Sciences, Nanchang University Medical College Nanchang, China

**Keywords:** CART, cocaine addiction, CaMKII, GABA_B_R, D_3_R

## Abstract

Cocaine- and amphetamine-regulated transcript (CART) peptides are neuropeptides that are expressed in brain regions associated with reward, such as the nucleus accumbens (NAc), and play a role in cocaine reward. Injection of CART into the NAc can inhibit the behavioral effects of cocaine, and injecting CART into the ventral tegmental area (VTA) reduces cocaine-seeking behavior. However, the exact mechanism of these effects is not clear. Recent research has demonstrated that Ca^2+^/calmodulin-dependent protein kinase II (CaMKII) and inhibitory G-protein coupled receptor (GPCR) signaling are involved in the mechanism of the effect of CART on cocaine reward. Hence, we review the role of CaMKII and inhibitory GPCR signaling in the effect of CART on cocaine reward and provide a new insight into the mechanism of that effect. In this article, we will first review the biological function of CART and discuss the role of CART in cocaine reward. Then, we will focus on the role of CaMKII and inhibitory GPCR signaling in cocaine reward. Furthermore, we will discuss how CaMKII and inhibitory GPCR signaling are involved in the mechanistic action of CART in cocaine reward. Finally, we will provide our opinions regarding the future directions of research on the role of CaMKII and inhibitory GPCR signaling in the effect of CART on cocaine reward.

## Introduction

Cocaine is a strong psychostimulant drug that can inhibit the reuptake of serotonin, norepinephrine and dopamine (DA). This results in greater concentrations of these three neurotransmitters in the brain. The drug can easily cross the blood-brain barrier and cause the user to feel intense euphoria (Pomara et al., 2012). In 2014, 18.3 million people were using cocaine worldwide. Cocaine can be administered by smoking, intravenous injection or inhalation, all of which can produce intense euphoric effects (United Nations Office on Drug and Crime (UNODC), 2016). Unfortunately, this euphoria can lead to increased frequency of use and dosage to obtain the same effect and to avoid the uncomfortable physiological and psychological effects linked to the cessation of drug use (Hou et al., 2014). Long-term and repeated cocaine use harms human health and contributes to crime, which places a great burden on families and society. However, the problem of cocaine abuse continues to spread. Therefore, there is an urgent need for new treatments to control and reduce the harm of cocaine abuse.

Cocaine- and amphetamine-regulated transcript (CART) is a neuropeptide that is expressed in brain regions associated with reward, such as the nucleus accumbens (NAc). During the last two decades, increasing evidence has demonstrated that CART plays a role in cocaine reward (Table 1). First, high densities of CART-containing nerve terminals are localized in brain regions associated with reward (Fagergren and Hurd, 2007). Second, injection of CART into the NAc can attenuate the behavioral effects of DA and cocaine (Hubert et al., 2008). Third, injection of CART into the paraventricular thalamus (PVT) can suppress cocaine-seeking behavior in rats (James et al., 2010). However, the mechanism by which CART inhibits the behavioral effect of DA is still not clear. Recently, some research has shown that Ca^2+^/calmodulin-dependent protein kinase II (CaMKII) and inhibitory G-protein coupled receptor (GPCR) signaling are involved in the mechanism of the effect of CART on cocaine reward, which may explain how CART inhibits the behavioral effect of DA and may help establish a more comprehensive mechanistic model of the effect of CART on cocaine reward. In the following sections, we will first review the role of CART in cocaine reward and note the shortcomings of the currently proposed mechanism for the effect of CART on cocaine reward. Then, we will focus on the role of CaMKII and inhibitory GPCR signaling in cocaine reward. In addition, we will discuss how CaMKII and inhibitory GPCR signaling are involved in the mechanism of the effect of CART on cocaine reward. Finally, we will provide our opinions regarding the future directions of research on the role of CaMKII and inhibitory GPCR signaling in the effect of CART on cocaine reward.

**Table 1:** The evidence that cocaine- and amphetamine-regulated transcript (CART) plays an important role in cocaine reward.

Species	Methods	Results	Mechanisms	References
Rat	Cocaine administration	CART expression ↑ in brain regions associated with reward	cAMP/PKA/CREB	Lakatos et al. (2002) and Cho et al. (2017)
Rat	Intra-NAc CART	Locomotor activity ↓	Inhibits the influx of Ca^2+^	Jaworski et al. (2003) and Peng et al. (2014)
Rat	Intra-NAc CART	Self-administration ↓	Ca^2+^/CaMKII, D3R	Jaworski et al. (2003) and Jaworski et al. (2008)
Rat	Intra-paraventricular thalamus CART	Cocaine-seeking behavior ↓	Unclear	James et al. (2010)
Monkey	Cocaine administration	CART expression ↑ in brain regions associated with reward	cAMP/PKA/CREB	Nader et al. (2002)
Human	Genetic studies	Contributed to the etiology of cocaine dependence	Unclear	Lohoff et al. (2008)

## Roles of CART in Cocaine Reward

### The Biological Features of CART

Douglass et al. (1995) used differential display PCR to screen for specific mRNAs that are transcriptionally regulated by cocaine and amphetamine in specific brain regions in rats. The authors identified a previously uncharacterized mRNA that was extracted from the hypothalamus by Spiess et al. (1981) and named it CART (Zhang et al., 2012). In addition to the brain, CART is also expressed in the pituitary gland, adrenal medulla and pancreas in humans. The CART peptide contains 116 amino acids encoded by the *cart* gene, which is located on the 5th chromosome in humans (Robson et al., 2002). The *cart* gene is approximately 2 kbp in length and contains two introns and three exons. There is a cyclic adenosine monophosphate (cAMP) response element (CRE) in the start codon of the *cart* gene (Perry Barrett et al., 2002). Therefore, CART could be upregulated by cocaine through the cAMP/protein kinase A (PKA)/CRE binding protein (CREB) signaling pathway. In addition to humans, the CART peptide has been found in other species, such as goldfish and mouse (Volkoff and Peter, 2001; Zhang et al., 2012). However, compared with humans, there are two alternatively spliced variants within exon 2 of rlCART. In goldfish, there are also two CART peptides. These two CART peptides originate not from alternative splicing but from the expression of two CART genes (Volkoff and Peter, 2001). Meanwhile, the sequence of the rat CART gene shows great homology with the human gene (Zhang et al., 2012). Many studies have demonstrated that the CART peptide can attenuate the behavioral effects of DA and cocaine and plays an important role in reward and reinforcement. In addition, the CART peptide is involved in feeding, stress and the regulation of the endocrine system (Asakawa et al., 2001; Kong et al., 2003; Larsen et al., 2003; Kuriyama et al., 2004; Koylu et al., 2006).

### CART Abnormalities in Cocaine Administration

CART mRNA has been found to be upregulated by cocaine or amphetamine in the rat striatum (Volkoff and Peter, 2001). However, this result has not been consistently replicated (Vrang et al., 2002). Hunter et al. (2005) have demonstrated that chronic and acute administration of cocaine failed to upregulate the levels of CART mRNA or peptide, but binge administration of cocaine resulted in increased CART mRNA in the NAc. The involvement of corticosterone may account for the abovementioned inconsistent results (Hunter et al., 2005). Binge administration of cocaine could cause significant stress to the rats, and corticosteroids are involved in stress. Meanwhile, Hunter et al. (2005) found that corticosterone administration produced a significant increase in CART mRNA, which suggests that CART mRNA may be regulated by cocaine under certain conditions, such as binge administration, and this may at least partly involve corticosterone. As estradiol may interact with the DA system, there is also a sex difference in the effect of cocaine on CART. Fagergren and Hurd (1999) found that CART levels were elevated in the medial accumbens shell and the central amygdala of male but not female rats after the administration of cocaine (Rodrigues et al., 2011). Additionally, this research showed that CART mRNA expression did not exhibit marked alterations in specific regions of the rat brain during the early phase of cocaine self-administration (Rodrigues et al., 2011). Furthermore, CART expression levels were increased in the NAc of human cocaine abusers (Albertson et al., 2004; Bannon et al., 2005).

### The Effect of CART on Cocaine Reward

**Figure 1:**
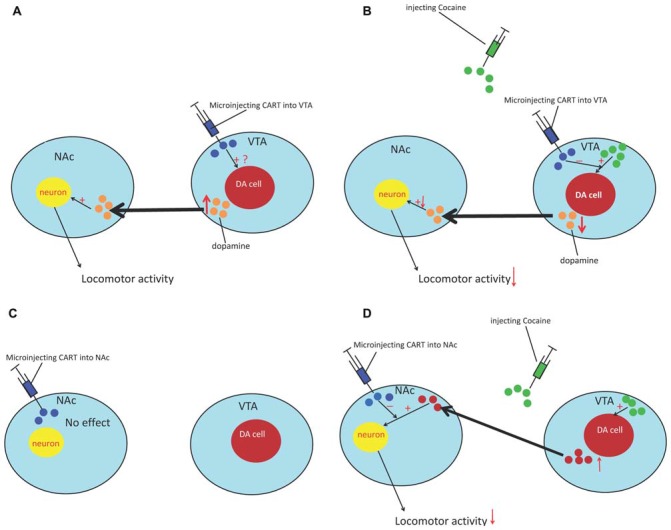
The effect of cocaine- and amphetamine-regulated transcript (CART) on cocaine reward. **(A)** Microinjecting the CART peptide into the ventral tegmental area (VTA) causes an efflux of DA in the NAc and induces locomotor activity (LMA). **(B)** Pretreatment of the VTA with CART can decrease the efflux of DA in the NAc and attenuate the locomotor effect induced by cocaine. **(C)** Microinjection of CART into the NAc had no effect. **(D)** Pretreatment with the CART peptide can reduce the sensitization of neuron response to DA and attenuate the locomotor-inducing effects of cocaine in the NAc. NAc, nucleus accumbens; VTA, ventral tegmental area; DA, dopamine.

Injection of CART peptide into the accumbens had no effect on locomotion (Kuhar et al., 2005). However, intra-accumbal CART attenuated the locomotor activity (LMA) produced by systemic cocaine and amphetamine administration. As cocaine induces LMA by potentiating dopaminergic transmission, the effect of CART 55-102 on DA-induced LMA was examined to explain the abovementioned phenomenon. As expected, CART peptide dose-dependently attenuated locomotion produced by intra-accumbal infusions of DA. These studies suggested that CART attenuates cocaine-induced LMA by attenuating the behavioral effect of DA. Meanwhile, intra-ventral tegmental area (VTA) injection of CART induced LMA and promoted conditioned place preference. The effect on LMA was dose dependent and was blunted by a DA receptor antagonist (Kimmel et al., 2000) However, pretreatment of the VTA with CART attenuated the locomotor effect induced by cocaine administration (Jaworski et al., 2007). Regarding the different results in the NAc and VTA, the potential mechanism may involve the cell bodies of the mesolimbic system that are located in the VTA; CART can produce weak psychostimulant-like effects and causes an efflux of DA in the NAc, which produces weak LMA (Kuhar et al., 2005) and suggests that CART can activate the DA system, but not in the same manner as cocaine (Figure 1). Meanwhile, this inhibitory effect of CART peptide also generalizes to other measures of dopaminergic function such as reward/reinforcement. Jaworski et al. (2008) found that injecting CART into the NAc could reduce cocaine self-administration in rats. Furthermore, James et al. (2010) reported that injection of CART into the PVT could suppress cocaine-seeking behavior in rats (James et al., 2010). However, the mechanisms through which CART influences the DA system are still unclear. Therefore, in the following sections, we provide a putative mechanism that involves CaMKII and inhibitory GPCR signaling to explain this abovementioned phenomenon.

## Roles of CaMKII in Cocaine Reward

### The Biological Features of CaMKII

CaMKII is a holoenzyme composed of subunits expressed by four genes (α, β, γ and δ; Rosenberg et al., 2010). This enzyme is abundant in brain cells, especially at the synapse. In the rat forebrain, there are two isoforms of CaMKIIα and CaMKIIβ. The functions and expression features of CaMKIIα and CaMKIIβ are different; CaMKIIα is expressed specifically in glutamatergic neurons (Liu and Murray, 2012), and the activity of αCaMKII is vital for memory formation and synaptic plasticity (Yamagata et al., 2009). CaMKIIβ is distributed in inhibitory interneurons and glutamatergic neurons (Lamsa et al., 2007). Activated CaMKII can phosphorylate its own autophosphorylation site (T286 in the isoform). Thus, CaMKII can convey information by diverse forms of Ca^2+^ transients and serve as a dynamic regulator that converts activity-dependent Ca^2+^ signals into different forms of plasticity and synaptic activity (Hudmon and Schulman, 2002; Colbran and Brown, 2004; Griffith, 2004). Recently, many studies have demonstrated that Ca^2+^ and Ca^2+^-regulated second messenger systems are involved in the behavioral response to cocaine in animals, and CaMKII plays an important role in the behavioral response to cocaine (Licata et al., 2004; Miller and Marshall, 2004).

### CaMKII and Cocaine Behavioral Sensitization

Cocaine can block the reuptake of DA and result in the accumulation of DA in the synaptic cleft. The accumulated DA can then promote glutamate release, which activates NMDA receptors and causes Ca^2+^ influx through NMDA receptors as well as L-type Ca^2+^ channels by activating D_1_ receptors and desensitizing D_3_ receptors (Wakabayashi and Kiyatkin, 2012). The influx of Ca^2+^ causes the activation of CaMKII, which promotes the phosphorylation of various targets and produces different biological effects, such as promoting the influx of Ca^2+^ and producing locomotion (Easton et al., 2014). Previous studies have shown that injecting an L-type Ca^2+^ channel antagonist can inhibit the expression of a sensitized behavioral response to amphetamine or cocaine (Park et al., 2001; Mills et al., 2007). Meanwhile, a great deal of research has shown that the overexpression of αCaMKII promotes behavioral sensitization to cocaine. Furthermore, injection of a CaMKII inhibitor (KN-93) into the VTA blunts the behavior sensitization produced by cocaine; consistent with KN-93 findings, behavioral sensitization to cocaine was attenuated in αCaMKII knockdown mice (Licata et al., 2004; Zhen et al., 2007; Kadivar et al., 2014). Together, these data demonstrate that CaMKII activity induced by the influx of Ca^2+^ can regulate behavioral sensitization to cocaine.

### CaMKII and Cocaine-Associated Memories

Individuals frequently encounter environmental cues previously associated with drug use that can increase craving and the likelihood of relapse (Fuchs et al., 2009; Kalivas, 2009). The ability of drug-associated memories to induce relapse is perhaps the greatest obstacle to the successful treatment of addictive disorders. Previous work has revealed that a single cocaine exposure can induce neuronal activation and long-term potentiation (LTP) in the VTA (the phosphorylation of CaMKII is very important for the induction of LTP; Ungless et al., 2001). Meanwhile, cocaine administration can increase the phosphorylation of CaMKII, and intra-VTA inhibition of CaMKII before cocaine conditioning blocks the acquisition of cocaine conditioned place preference (Liu et al., 2014; Schöpf et al., 2015). Together, those results suggest that CaMKII plays an important role in the formation of cocaine-associated memories. Furthermore, Rich et al. (2016) found that intra-basolateral amygdala inhibition of CaMKII promoted the extinction of cocaine-associated memory and led to a reduction in subsequent cue-induced reinstatement, which provides a novel target for preventing relapse to cocaine use.

## Roles of Inhibitory G-Protein Coupled Receptor Signaling in Cocaine Reward

### The Biological Features of Inhibitory G-Protein Coupled Receptor Signaling

**Figure 2:**
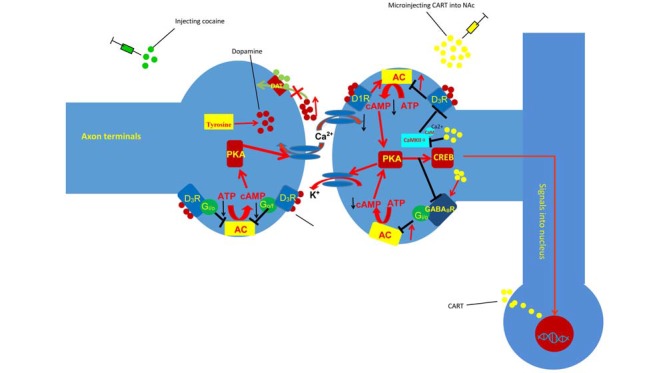
Injection of CART blunts the effect of cocaine on inhibitory G-protein coupled receptor (GPCR) signaling and Ca^2+^/calmodulin-dependent protein kinase II (CaMKII). Cocaine can block the reuptake of DA and result in the accumulation of DA in the synaptic cleft. The accumulated DA can decrease the sensitivity of the D3 DA autororeceptors located on dopaminergic cells and favor somatodendritic DA release. Conversely, DA activates D1 DA heteroreceptors, desensitizes the D3 DA autoreceptors located on dopaminergic cells, and stimulates AC, resulting in increased intracellular cAMP levels. However, injecting CART into the NAc decreases the phosphorylation of CaMKIIα and D3R, which inhibits the activity of AC and reduces cocaine-induced LMA. D_1_R, dopamine D1 receptor; D_3_R, dopamine D3 receptor; PKA, protein kinase A; ATP, adenosine triphosphate; cAMP, cyclic adenosine monophosphate; AC, adenylyl cyclase; DAT, dopamine transporter; CREB, cAMP-response element binding protein.

GPCRs are seven-transmembrane-domain receptors, which transduce ligand-binding events into intracellular responses. Depending on the α subunit type, GPCRs can be Gαs, Gαi/o, Gαq/11 or Gα12/13 (Wettschureck and Offermanns, 2005; Oldham and Hamm, 2008). Gαi/o can interact with downstream effectors and inhibit excitatory effectors, including most isoforms of adenylyl cyclase (AC) and some types of voltage-gated Ca^2+^ channels. Metabotropic γ-aminobutyric acid receptors (GABA_B_Rs) and D_3_ receptors couple to and activate the Gi/o subclass of Gα subunits and produce inhibitory signaling (Filip et al., 2007). Currently, increasing evidence has indicated that inhibitory GPCR signaling mediated by the Gi/o class of GPCRs for the neurotransmitters GABA (GABA_B_R) and DA (D_3_ receptors) plays an important role in cocaine reward (Goldstein and Volkow, 2002; Vlachou and Markou, 2010; Figure 2). Many studies have shown that GABA_B_R, D_3_R and CART are co-expressed in some brain regions, such as the NAc (Liu et al., 2009; Hubert et al., 2010; Fu et al., 2016). Meanwhile, some research has demonstrated that GABA_B_R and D3Rs can interact with CART (Hubert et al., 2010; Peng et al., 2014; Fu et al., 2016). Therefore, GABA_B_Rs and D3Rs, as representative inhibitory GPCR signaling are chosen to illustrate the involvement of inhibitory GPCR signaling in the role of CART in cocaine reward.

### GABA_B_R and Cocaine Reward

GABA_B_Rs are metabotropic receptors that belong to the GPCR superfamily and are responsible for the neuromodulation of GABA (Yamaguchi et al., 2002). The GABA_B_Rs are distributed at pre- and post-synaptic sites. The activation of presynaptic GABA_B_Rs can block calcium channels. The activation of post-synaptic GABA_B_Rs activates potassium channels and increases the flux of potassium from extracellular to intracellular sites. Chronic cocaine administration decreases the functional coupling of GABA_B_ receptors in the rat brain (Kushner and Unterwald, 2001; Jayaram and Steketee, 2004). Meanwhile, the GABA_B_R agonist baclofen attenuates cocaine-induced hyperlocomotion, and intra-VTA application of baclofen blunts cocaine self-administration (Brebner et al., 2000). Furthermore, clinical research has shown that baclofen can attenuate cue-associated cocaine craving and reduce cocaine use in a double-blind placebo-controlled trial (Shoptaw et al., 2003).

### D_3_R and Cocaine Reward

D_3_Rs are GPCRs and belong to the class of D_2_-like receptors, which can inhibit AC and negatively modulate the activity of PKA and its effectors (Rangel-Barajas et al., 2015). In humans, D_3_Rs are expressed in the islands of Calleja, ventral striatum/NAc, dentate gyrus and striate cortex (Heidbreder et al., 2005). Compared with D_2_ receptors, D_3_Rs have a high affinity for DA. Small changes in their number or function may lead to dramatic effects on synaptic transmission, suggesting that D_3_ receptors could be critical for modulating dopaminergic function. Many studies found that a D_3_R agonist (BP897) blunted cocaine-seeking behavior during the presentation of cocaine-associated cues in rats (Cervo et al., 2003; Gilbert et al., 2005; Cortés et al., 2016). Furthermore, increasing evidence has shown that selective D_3_ receptor agonists can attenuate cocaine self-administration (Beardsley et al., 2001; Cheung et al., 2013). Altogether, these studies indicate that D_3_Rs play a vital role in cocaine addiction and may be a potential target for drug treatments.

## The Interaction of CART with CaMKII and D_3_R After Repeated Cocaine Administration

Repeated cocaine administration decreases the sensitivity of the D_3_ DA autororeceptors located on dopaminergic cells and reduces G-protein-mediated K^+^ efflux, which favors somatodendritic DA release (Licata and Pierce, 2003). Conversely, DA activates D_1_ DA heteroreceptors, desensitizes the D_3_ DA autoreceptors located on dopaminergic cells, and stimulates AC, resulting in increased intracellular cAMP levels (Licata and Pierce, 2003). The increased intracellular cAMP levels can then activate PKA and ultimately upregulate the expression of CART via the cAMP/PKA/CREB signaling pathway (Lakatos et al., 2002). Meanwhile, the Ca^2+^ influx induced by cocaine can activate CaMKII. Furthermore, the autophosphorylation of CaMKIIα(T286) by Ca^2+^/CaM could enhance the binding of CaMKIIα to D3Rs, which further stimulates the phosphorylation of D3Rs and transiently inhibits the efficacy of those receptors (Liu et al., 2009). Hence, the influx of Ca^2+^ induced by cocaine can stimulate CaMKII, which inhibits the activation of D_3_R (Avalos-Fuentes et al., 2015). Furthermore, previous studies have shown that injection of CART into the NAc can decrease the phosphorylation of CaMKIIα on threonine (T) 286 and D_3_R (Peng et al., 2014; Fu et al., 2016), which demonstrates that CART can inhibit the activation of CaMKII and further favor the activation of D_3_R (Figure 2).

## The Putative Mechanism by Which CART Attenuates The Behavioral Effect of Dopamine

The influx of DA in the NAc activates the D_1_ DA heteroreceptors and desensitizes the D_3_ DA autoreceptors, which activate downstream targets, such as CREB expression and Ca^2+^ signaling, and produce LMA. Previous studies have shown that CART attenuates the behavioral effect of cocaine by inhibiting the behavioral effect of DA. However, the mechanisms through which CART inhibits the behavioral effect of DA are not clear. Some research has shown that injecting CART into the NAc decreases the phosphorylation of CaMKIIα on threonine (T) 286 and D_3_R and reduces cocaine-induced LMA (Peng et al., 2014; Fu et al., 2016). Meanwhile, research has demonstrated that injection of the CaMKIIα inhibitor KN-93 into the NAc attenuates cocaine-enhanced locomotion (Kadivar et al., 2014). Together, those results suggest that CART inhibits Ca^2+^ signaling and attenuates the behavioral effect of DA by reducing the phosphorylation of CaMKII on threonine (T) 286. Specifically, activated CaMKIIα can stimulate the phosphorylation of D_3_Rs and transiently inhibit D_3_R efficacy (Liu et al., 2009). Meanwhile, D_3_Rs could couple with Gi/o proteins and inhibit the cAMP/PKA cascade (Avalos-Fuentes et al., 2015). Considered together, the evidence suggests that CART can inhibit the activation of CaMKIIα and further promote the activation of D_3_Rs, which reduce the sensitization response to DA and attenuate the behavioral effect of the neurotransmitter (Figure 2).

## Future Studies

Since CART was first found to be upregulated by cocaine administration, increasing attention has been paid to the role of CART in cocaine reward (Zhang et al., 2012). Subsequently, other studies have shown that injecting CART into the NAc can attenuate cocaine-induced LMA (Jaworski et al., 2003). By way of explaining this phenomenon further, injecting DA into the NAc results in an increase in LMA, and injecting both DA and CART into the NAc reduces the effect of DA (Kuhar et al., 2005). These findings suggest that CART opposes the actions of cocaine by influencing DA in the NAc. Our previous research has shown that injecting CART into the NAc reduces cocaine-induced LMA by decreasing the phosphorylation of D_3_R and the expression of pCaMKIIα (Fu et al., 2016). This result further demonstrates that cocaine-induced LMA also involves CaMKII and D_3_R. Furthermore, increasing studies show that GABA_B_R may also involve in the mechanistic action of CART in cocaine reward. Previous studies have shown that CART peptides are present in a subset of GABAergic projection neurons that express dynorphin, which inhibits the effect of DA by activating the κ-receptor in the NAc (Dallvechia-Adams et al., 2002; Hubert and Kuhar, 2006). Moreover, CART-containing axons and nerve terminals activate dopaminergic neurons, with some CART peptide-positive terminals forming inhibitory synapses onto GABAergic interneurons in the VTA and substantia nigra (SN; Dallvechia-Adams et al., 2001, 2002). Coincidentally, a previous report (Hubert et al., 2010) has shown that CART-containing terminals that originate in the NAc form symmetric synapses onto inhibitory GABAergic synapses and inhibit cocaine-induced locomotion in the ventral pallidum. These studies suggest that there are functional associations between CART and the GABAergic system (Bäckberg et al., 2003). Meanwhile, some studies have shown that GABA_B_R and CART are highly expressed in NAc (Filip and Frankowska, 2008; Hubert et al., 2010; Fu et al., 2016). Furthermore, injecting CART into the NAc increases GABA_B_R expression (data not shown). In addition, baclofen (GABA_B_ receptor agonist) antagonizes cocaine-induced DA release in the NAc and blocks cocaine-induced hyperlocomotion (Filip et al., 2015). Considered together, the evidence suggests that CART may attenuate cocaine-induced locomotion by influencing the expression of GABA_B_R. However, further investigations are needed to determine the mechanism by which CART exerts its effect on cocaine-induced locomotion.

Compulsive drug-taking behavior and high rates of relapse are the two main characteristics of drug addiction. Relapse is a difficult problem to solve in drug addiction treatment and occurs when the drug-seeking habit is reactivated by drug-related cues. Initially, the addict may retrieve from memory strongly compelling drug-related experiences, which then lead to further drug-seeking and drug-taking behavior. Currently, research on the effect of CART on cocaine reward mainly focuses on LMA. There are only a few studies on the effect of CART on relapse. James et al. (2010) found that injecting CART into the PVT attenuates cocaine-seeking behavior in rats. However, the mechanism underlying the inhibitory effect of CART on cocaine-seeking behavior remains unclear. Meanwhile, decreased cocaine-seeking behavior induced by CART may involve D_3_R and GABA_B_R. D_3_R agonists and GABA_B_R agonists can inhibit cocaine-seeking behavior (Beardsley et al., 2001; Cheung et al., 2013; Blacktop et al., 2016). Furthermore, injecting CART into the NAc decreases the phosphorylation of D_3_R (Fu et al., 2016) and increases GABA_B_R expression (data not shown). The totality of the evidence suggests that CART not only reduces the rewarding effects of cocaine by inhibiting cocaine self-administration patterns but also prevents relapse to cocaine use; therefore, it represents an important potential target for drug treatments.

## Author Contributions

CY and XZ wrote the manuscript. ZH provided the idea for this work and designed the structure of the manuscript. The other authors revised the manuscript.

## Conflict of Interest Statement

The authors declare that the research was conducted in the absence of any commercial or financial relationships that could be construed as a potential conflict of interest.

## References

[B1] Albertson D. N. Pruetz B. Schmidt C. J. Kuhn D. M. Kapatos G. Bannon M. J. (2004). Gene expression profile of the nucleus accumbens of human cocaine abusers:evidence for dysregulation of myelin. J. Neurochem. 88, 1211–1219. 10.1046/j.1471-4159.2003.02247.x 15009677 PMC2215309

[B2] Asakawa A. Inui A. Yuzuriha H. Nagata T. Kaga T. Ueno N.. (2001). Cocaine-amphetamine-regulated transcript influences energy metabolism, anxiety and gastric emptying in mice. Horm. Metab. Res. 33, 554–558. 10.1055/s-2001-17205 11561216

[B3] Avalos-Fuentes A. Albarrán-Bravo S. Loya-Lopéz S. Cortés H. Recillas-Morales S. Magaña J. J.. (2015). Dopaminergic denervation switches dopamine d3 receptor signaling and disrupts its Ca^2+^ dependent modulation by camkii and calmodulin in striatonigral projections of the rat. Neurobiol. Dis. 74, 336–346. 10.1016/j.nbd.2014.12.008 25517101

[B4] Bäckberg M. Collin M. Ovesjö M. L. Meister B. (2003). Chemical coding of GABA_B_ receptor-immunoreactive neurones in hypothalamic regions regulating body weight. J. Neuroendocrinol. 15, 1–14. 10.1046/j.1365-2826.2003.00843.x 12535164

[B5] Bannon M. Kapatos G. Albertson D. (2005). Gene expression profiling in the brains of human cocaine abusers. Addict. Biol. 10, 119–126. 10.1080/13556210412331308921 15849025 PMC2215307

[B6] Beardsley P. M. Sokoloff P. Balster R. L. Schwartz J.-C. (2001). The D3R partial agonist, BP 897, attenuates the discriminative stimulus effects of cocaine and D-amphetamine and is not self-administered. Behav. Pharmacol. 12, 1–11. 10.1097/00008877-200102000-00001 11270507

[B7] Blacktop J. M. Vranjkovic O. Mayer M. Van Hoof M. Baker D. A. Mantsch J. R. (2016). Antagonism of GABA-B but not GABA-A receptors in the VTA prevents stress- and intra-VTA CRF-induced reinstatement of extinguished cocaine seeking in rats. Neuropharmacology 102, 197–206. 10.1016/j.neuropharm.2015.11.013 26596556 PMC4717150

[B9] Brebner K. Phelan R. Roberts D. C. (2000). Intra-VTA baclofen attenuates cocaine self-administration on a progressive ratio schedule of reinforcement. Pharmacol. Biochem. Behav. 66, 857–862. 10.1016/s0091-3057(00)00286-0 10973526

[B62] Cervo L. Carnovali F. Stark J. A. Mennini T. (2003). Cocaine-seeking behavior in response to drug-associated stimuli in rats: involvement of D_3_ and D_2_ dopamine receptors. Neuropsychopharmacology 28, 1150–1159. 10.1038/sj.npp.1300169 12700684

[B10] Cheung T. H. Loriaux A. L. Weber S. M. Chandler K. N. Lenz J. D. Schaan R. F.. (2013). Reduction of cocaine self-administration and D3 receptor-mediated behavior by two novel dopamine D3 receptor-selective partial agonists, OS-3–106 and WW-III-55. J. Pharmacol. Exp. Ther. 347, 410–423. 10.1124/jpet.112.202911 24018640 PMC3807071

[B11] Cho B. R. Yoon H. S. Kim W. Y. Vezina P. Kim J. H. (2017). Cocaine inhibits leptin-induced increase of the cocaine- and amphetamine-regulated transcript peptide in the nucleus accumbens in rats. Neuroreport 28, 701–704. 10.1097/WNR.0000000000000822 28614180

[B12] Colbran R. J. Brown A. M. (2004). Calcium/calmodulin-dependent protein kinase ii and synaptic plasticity. Curr. Opin. Neurobiol. 14, 318–327. 10.1016/s0959-4388(04)00075-3 15194112

[B13] Cortés A. Moreno E. Rodríguez-Ruiz M. Canela E. I. Casadó V. (2016). Targeting the dopamine D_3_ receptor: an overview of drug design strategies. Expert Opin. Drug Discov. 11, 641–664. 10.1080/17460441.2016.1185413 27135354

[B14] Dallvechia-Adams S. Kuhar M. J. Smith Y. (2002). Cocaine- and amphetamine-regulated transcript peptide projections in the ventral midbrain: colocalization with γ-aminobutyric acid, melanin-concentrating hormone, dynorphin, and synaptic interactions with dopamine neurons. J. Comp. Neurol. 448, 360–372. 10.1002/cne.10268 12115699

[B15] Dallvechia-Adams S. Smith Y. Kuhar M. J. (2001). Cart peptide-immunoreactive projection from the nucleus accumbens targets substantia nigra pars reticulata neurons in the rat. J. Comp. Neurol. 434, 29–39. 10.1002/cne.1162 11329127

[B16] Douglass J. McKinzie A. A. Couceyro P. (1995). PCR differential display identifies a rat brain mRNA that is transcriptionally regulated by cocaine and amphetamine. J. Neurosci. 15, 2471–2481. 7891182 10.1523/JNEUROSCI.15-03-02471.1995 PMC6578117

[B17] Easton A. C. Lourdusamy A. Havranek M. Mizuno K. Solati J. Golub Y.. (2014). αCaMKII controls the establishment of cocaine’s reinforcing effects in mice and humans. Transl. Psychiatry 4:e457. 10.1038/tp.2014.97 25290264 PMC4350526

[B19] Fagergren P. Hurd Y. L. (1999). Mesolimbic gender differences in peptide CART mRNA expression: effects of cocaine. Neuroreport 10, 3449–3452. 10.1097/00001756-199911080-00034 10599860

[B20] Fagergren P. Hurd Y. (2007). CART mRNA expression in rat monkey and human brain: relevance to cocaine abuse. Physiol. Behav. 92, 218–225. 10.1016/j.physbeh.2007.05.027 17631364

[B21] Filip M. Frankowska M. (2008). GABA_B_ receptors in drug addiction. Pharmacol. Rep. 60, 755–770. 19211967

[B22] Filip M. Frankowska M. Przegaliński E. (2007). Effects of GABA_B_ receptor antagonist, agonists and allosteric positive modulator on the cocaine-induced self-administration and drug discrimination. Eur. J. Pharmacol. 574, 148–157. 10.1016/j.ejphar.2007.07.048 17698060

[B23] Filip M. Frankowska M. Sadakierska-Chudy A. Suder A. Szumiec L. Mierzejewski P.. (2015). GABA_B_ receptors as a therapeutic strategy in substance use disorders: focus on positive allosteric modulators. Neuropharmacology 88, 36–47. 10.1016/j.neuropharm.2014.06.016 24971600

[B24] Fu Q. Zhou X. Dong Y. Huang Y. Yang J. Oh K. W.. (2016). Decreased caffeine-induced locomotor activity via microinjection of cart peptide into the nucleus accumbens is linked to inhibition of the pcamkiia-d3r interaction. PLoS One 11:e0159104. 10.1371/journal.pone.0159104 27404570 PMC4942143

[B25] Fuchs R. A. Bell G. H. Ramirez D. R. Eaddy J. L. Su Z. I. (2009). Basolateral amygdala involvement in memory reconsolidation processes that facilitate drug context-induced cocaine seeking. Eur. J. Neurosci. 30, 889–900. 10.1111/j.1460-9568.2009.06888.x 19712099 PMC2759304

[B27] Gilbert J. G. Newman A. H. Gardner E. L. Ashby C. R. Jr. Heidbreder C. A. Pak A. C.. (2005). Acute administration of SB-277011A, NGB 2904, or BP 897 inhibits cocaine cue-induced reinstatement of drug-seeking behavior in rats: role of dopamine D_3_ receptors. Synapse 57, 17–28. 10.1002/syn.20152 15858839 PMC3726034

[B28] Goldstein R. Z. Volkow N. D. (2002). Drug addiction and its underlying neurobiological basis: neuroimaging evidence for the involvement of the frontal cortex. Am. J. Psychiatry 159, 1642–1652. 10.1176/appi.ajp.159.101642 12359667 PMC1201373

[B29] Griffith L. C. (2004). Regulation of calcium/calmodulin-dependent protein kinase ii activation by intramolecular and intermolecular interactions. J. Neurosci. 24, 8394–8398. 10.1523/JNEUROSCI.3604-04.2004 15456810 PMC6729912

[B30] Heidbreder C. A. Gardner E. L. Xi Z.-X. Thanos P. K. Mugnaini M. Hagan J. J.. (2005). The role of central dopamine D_3_ receptors in drug addiction: a review of pharmacological evidence. Brain Res. Rev. 49, 77–105. 10.1016/j.brainresrev.2004.12.033 15960988 PMC3732040

[B31] Hou H. Wang C. Jia S. Hu S. Tian M. (2014). Brain dopaminergic system changes in drug addiction: a review of positron emission tomography findings. Neurosci. Bull. 30, 765–776. 10.1007/s12264-014-1469-5 25260796 PMC5562592

[B33] Hubert G. W. Jones D. C. Moffett M. C. Rogge G. Kuhar M. J. (2008). Cart peptides as modulators of dopamine and psychostimulants and interactions with the mesolimbic dopaminergic system. Biochem. Pharmacol. 75, 57–62. 10.1016/j.bcp.2007.07.028 17854774 PMC3804336

[B32] Hubert G. W. Kuhar M. J. (2006). Colocalization of cart peptide with prodynorphin and dopamine d1 receptors in the rat nucleus accumbens. Neuropeptides 40, 409–415. 10.1016/j.npep.2006.09.001 17064765

[B34] Hubert G. W. Manvich D. F. Kuhar M. J. (2010). Cocaine and amphetamine-regulated transcript-containing neurons in the nucleus accumbens project to the ventral pallidum in the rat and may inhibit cocaine-induced locomotion. Neuroscience 165, 179–187. 10.1016/j.neuroscience.2009.10013 19825396 PMC3804330

[B35] Hudmon A. Schulman H. (2002). Neuronal Ca^2+^/calmodulin-dependent protein kinase II: the role of structure and autoregulation in cellular function. Annu. Rev. Biochem. 71, 473–510. 10.1146/annurev.biochem.71.110601.135410 12045104

[B36] Hunter R. G. Vicentic A. Rogge G. Kuhar M. J. (2005). The effects of cocaine on cart expression in the rat nucleus accumbens: a possible role for corticosterone. Eur. J. Pharmacol. 517, 45–50. 10.1016/j.ejphar.2005.05.025 15972209

[B37] James M. H. Charnley J. L. Jones E. Levi E. M. Yeoh J. W. Flynn J. R.. (2010). Cocaine- and amphetamine-regulated transcript (CART) signaling within the paraventricular thalamus modulates cocaine-seeking behaviour. PLoS One 5:e12980. 10.1371/journal.pone.0012980 20886038 PMC2944892

[B40] Jaworski J. N. Hansen S. T. Kuhar M. J. Mark G. P. (2008). Injection of cart (cocaine- and amphetamine-regulated transcript) peptide into the nucleus accumbens reduces cocaine self-administration in rats. Behav. Brain Res. 191, 266–271. 10.1016/j.bbr.2008.03.039 18485497 PMC2497435

[B38] Jaworski J. N. Kimmel H. L. Mitrano D. A. Tallarida R. J. Kuhar M. J. (2007). Intra-vta cart 55–102 reduces the locomotor effect of systemic cocaine in rats: an isobolographic analysis. Neuropeptides 41, 65–72. 10.1016/j.npep.2006.12.003 17289142 PMC1994245

[B39] Jaworski J. N. Kozel M. A. Philpot K. B. Kuhar M. J. (2003). Intra-accumbal injection of CART (cocaine-amphetamine regulated transcript) peptide reduces cocaine-induced locomotor activity. J. Pharmacol. Exp. Ther. 307, 1038–1044. 10.1124/jpet.103.052332 14551286

[B41] Jayaram P. Steketee J. D. (2004). Effects of repeated cocaine on medial prefrontal cortical GABA_B_ receptor modulation of neurotransmission in the mesocorticolimbic dopamine system. J. Neurochem. 90, 839–847. 10.1111/j.1471-4159.2004.02525.x 15287889

[B43] Kadivar M. Farahmandfar M. Ranjbar F. E. Zarrindast M. R. (2014). Increased calcium/calmodulin-dependent protein kinase ii activity by morphine-sensitization in rat hippocampus. Behav. Brain Res. 267, 74–82. 10.1016/j.bbr.2014.03.035 24675163

[B44] Kalivas P. W. (2009). The glutamate homeostasis hypothesis of addiction. Nat. Rev. Neurosci. 10, 561–572. 10.1038/nrn2515 19571793

[B45] Kimmel H. L. Gong W. Vechia S. D. Hunter R. G. Kuhar M. J. (2000). Intra-ventral tegmental area injection of rat cocaine and amphetamine-regulated transcript peptide 55–102 induces locomotor activity and promotes conditioned place preference. J. Pharmacol. Exp. Ther. 294, 784–792. 10900261

[B46] Kong W. Stanley S. Gardiner J. Abbott C. Murphy K. Seth A.. (2003). A role for arcuate cocaine and amphetamine-regulated transcript in hyperphagia, thermogenesis, and cold adaptation. FASEB J. 17, 1688–1690. 10.1096/fj.02-0805fje 12958177

[B48] Koylu E. O. Balkan B. Kuhar M. J. Pogun S. (2006). Cocaine and amphetamine regulated transcript (CART) and the stress response. Peptides 27, 1956–1969. 10.1016/j.peptides.2006.03.032 16822586

[B49] Kuhar M. J. Jaworski J. N. Hubert G. W. Philpot K. B. Dominguez G. (2005). Cocaine- and amphetamine-regulated transcript peptides play a role in drug abuse and are potential therapeutic targets. AAPS J. 7, E259–E265. 10.1208/aapsj070125 16146347 PMC2751515

[B50] Kuriyama G. Takekoshi S. Tojo K. Nakai Y. Kuhar M. J. Osamura R. Y. (2004). Cocaine- and amphetamine-regulated transcript peptide in the rat anterior pituitary gland is localized in gonadotrophs and suppresses prolactin secretion. Endocrinology 145, 2542–2550. 10.1210/en.2003-0845 14764627

[B51] Kushner S. A. Unterwald E. M. (2001). Chronic cocaine administration decreases the functional coupling of GABA_B_, receptors in the rat ventral tegmental area as measured by baclofen-stimulated ^35^S-GTPγS binding. Life Sci. 69, 1093–1102. 10.1016/s0024-3205(01)01203-6 11508652

[B52] Lakatos A. Dominguez G. Kuhar M. J. (2002). CART promoter CRE site binds phosphorylated CREB. Mol. Brain Res. 104, 81–85. 10.1016/s0169-328x(02)00321-2 12117553

[B53] Lamsa K. Irvine E. E. Giese K. P. Kullmann D. M. (2007). NMDA receptor-dependent long-term potentiation in mouse hippocampal interneurons shows a unique dependence on Ca^2+^/calmodulin-dependent kinases. J. Physiol. 584, 885–894. 10.1113/jphysiol.2007.137380 17884930 PMC2276991

[B54] Larsen P. J. Seier V. Fink-Jensen A. Holst J. J. Warberg J. Vrang N. (2003). Cocaine- and amphetamine-regulated transcript is present in hypothalamic neuroendocrine neurones and is released to the hypothalamic-pituitary portal circuit. J. Neuroendocrinol. 15, 219–226. 10.1046/j.1365-2826.2003.00960.x 12588509

[B55] Licata S. C. Pierce R. C. (2003). The roles of calcium/calmodulin-dependent and ras/mitogen-activated protein kinases in the development of psychostimulant-induced behavioral sensitization. J. Neurochem. 85, 14–22. 10.1046/j.1471-4159.2003.01662.x 12641723

[B56] Licata S. C. Schmidt H. D. Pierce R. C. (2004). Suppressing calcium/calmodulin-dependent protein kinase II activity in the ventral tegmental area enhances the acute behavioural response to cocaine but attenuates the initiation of cocaine-induced behavioural sensitization in rats. Eur. J. Neurosci. 19, 405–414. 10.1111/j.0953-816x.2003.03110x 14725635

[B60] Liu X. Liu Y. Zhong P. Wilkinson B. Qi J. Olsen C. M.. (2014). Camkii activity in the ventral tegmental area gates cocaine-induced synaptic plasticity in the nucleus accumbens. Neuropsychopharmacology 39, 989–999. 10.1038/npp.2013.299 24154664 PMC3924533

[B59] Liu X.-Y. Mao L.-M. Zhang G.-C. Papasian C. J. Fibuch E. E. Lan H. X.. (2009). Activity-dependent modulation of limbic dopamine D3 receptors by CaMKII. Neuron 61, 425–438. 10.1016/j.neuron.2008.12.015 19217379 PMC2650276

[B58] Liu X. B. Murray K. D. (2012). Neuronal excitability and calcium/calmodulin-dependent protein kinase type II: location, location, location. Epilepsia 53, 45–52. 10.1111/j.1528-1167.2012.03474.x 22612808

[B61] Lohoff F. W. Bloch P. J. Weller A. E. Nall A. H. Doyle G. A. Buono R. J.. (2008). Genetic variants in the cocaine- and amphetamine-regulated transcript gene (*CARTPT*) and cocaine dependence. Neurosci. Lett. 440, 280–283. 10.1016/j.neulet.2008.05.073 18572320 PMC2507865

[B63] Miller C. A. Marshall J. F. (2004). Altered prelimbic cortex output during cue-elicited drug seeking. J. Neurosci. 24, 6889–6897. 10.1523/JNEUROSCI.1685-04.2004 15295023 PMC6729607

[B64] Mills K. Ansah T. A. Ali S. F. Mukherjee S. Shockley D. C. (2007). Augmented behavioral response and enhanced synaptosomal calcium transport induced by repeated cocaine administration are decreased by calcium channel blockers. Life Sci. 81, 600–608. 10.1016/j.lfs.2007.06.028 17689567 PMC2765982

[B101] Nader M. A. Daunais J. B. Moore T. Nader S. H. Moore R. J. Smith H. R.. (2002). Effects of cocaine self-administration on striatal dopamine systems in rhesus monkeys: initial and chronic exposure. Neuropsychopharmacology 27, 35–46. 10.1016/S0893-133X(01)00427-4 12062905

[B65] Oldham W. M. Hamm H. E. (2008). Heterotrimeric G protein activation by G-protein-coupled receptors. Nat. Rev. Mol. Cell Biol. 9, 60–71. 10.1038/nrm2299 18043707

[B66] Park K. Vora U. Darling S. F. Kolta M. G. Soliman K. F. A. (2001). The role of inducible nitric oxide synthase in cocaine-induced locomotor sensitization. Physiol. Behav. 74, 441–447. 10.1016/s0031-9384(01)00588-1 11790403

[B67] Peng Q. Sun X. Liu Z. Yang J. Oh K. W. Hu Z. (2014). Microinjection of cart (cocaine- and amphetamine-regulated transcript) peptide into the nucleus accumbens inhibits the cocaine-induced upregulation of dopamine receptors and locomotor sensitization. Neurochem. Int. 75, 105–111. 10.1016/j.neuint.2014.06.005 24953280

[B68] Perry Barrett M. D. Davidson J. Morgan P. (2002). CART gene promoter transcription is regulated by a cyclic adenosine monophosphate response element. Obes. Res. 10, 1291–1298. 10.1038/oby.2002.175 12490674

[B70] Pomara C. Cassano T. D’Errico S. Bello S. Romano A. D. Riezzo I.. (2012). Data available on the extent of cocaine use and dependence: biochemistry, pharmacologic effects and global burden of disease of cocaine abusers. Curr. Med. Chem. 19, 5647–5657. 10.2174/092986712803988811 22856655

[B71] Rangel-Barajas C. Coronel I. Florán B. (2015). Dopamine receptors and neurodegeneration. Aging Dis. 6, 349–368. 10.14336/AD.2015.0330 26425390 PMC4567218

[B72] Rich M. T. Abbott T. B. Chung L. Gulcicek E. E. Stone K. L. Colangelo C. M.. (2016). Phosphoproteomic analysis reveals a novel mechanism of camkiiα regulation inversely induced by cocaine memory extinction versus reconsolidation. J. Neurosci. 36, 7613–7627. 10.1523/JNEUROSCI.1108-16.2016 27445140 PMC4951572

[B73] Robson A. J. Rousseau K. Loudon A. S. I. Ebling F. J. P. (2002). Cocaine and amphetamine-regulated transcript mrna regulation in the hypothalamus in lean and obese rodents. J. Neuroendocrinol. 14, 697–709. 10.1046/j.1365-2826.2002.00830.x 12213131

[B74] Rodrigues B. C. Cavalcante J. C. Elias C. F. (2011). Expression of cocaine- and amphetamine-regulated transcript in the rat forebrain during postnatal development. Neuroscience 195, 201–214. 10.1016/j.neuroscience.2011.08.036 21903152 PMC4235957

[B75] Rosenberg O. S. Deindl S. Sung R. J. Nairn A. C. Kuriyan J. (2010). Structure of the autoinhibited kinase domain of camkii and saxs analysis of the holoenzyme. Cell 123, 849–860. 10.1016/j.cell.2005.10029 16325579

[B76] Schöpf I. Easton A. C. Solati J. Golub Y. Kornhuber J. Giese K. P.. (2015). αCaMKII autophosphorylation mediates neuronal activation in the hippocampal dentate gyrus after alcohol and cocaine in mice. Neurosci. Lett. 591, 65–68. 10.1016/j.neulet.2015.02.031 25700946

[B78] Shoptaw S. Yang X. Rotheram-Fuller E. J. Hsieh Y. C. Kintaudi P. C. Charuvastra V. C.. (2003). Randomized placebo-controlled trial of baclofen for cocaine dependence: preliminary effects for individuals with chronic patterns of cocaine use. J. Clin. Psychiatry 64, 1440–1448. 10.4088/jcp.v64n1207 14728105

[B100] Spiess J. Villarreal J. Vale W. (1981). Isolation and sequence analysis of a somatostatin-like polypeptide from ovine hypothalamus. Biochemistry 20, 1982–1988. 10.1021/bi00510a038 7225368

[B82] Ungless M. A. Whistler J. L. Malenka R. C. Bonci A. (2001). Single cocaine exposure *in vivo* induces long-term potentiation in dopamine neurons. Nature 411, 583–587. 10.1038/35079077 11385572

[B800] United Nations Office on Drug and Crime (UNODC) (2016). World Drug Report. Available online at: http://www.unodc.org/wdr2016/

[B83] Vlachou S. Markou A. (2010). GABA_B_ receptors in reward processes. Adv. Pharmacol. 58, 315–371. 10.1016/s1054-3589(10)58013-x 20655488

[B84] Volkoff H. Peter R. E. (2001). Characterization of two forms of cocaine- and amphetamine-regulated transcript (CART) peptide precursors in goldfish: molecular cloning and distribution, modulation of expression by nutritional status, and interactions with leptin. Endocrinology 142, 5076–5088. 10.1210/endo.142.12.8519 11713200

[B85] Vrang N. Larsen P. J. Kristensen P. (2002). Cocaine-amphetamine regulated transcript (CART) expression is not regulated by amphetamine. Neuroreport 13, 1215–1218. 10.1097/00001756-200207020-00029 12151772

[B86] Wakabayashi K. T. Kiyatkin E. A. (2012). Rapid changes in extracellular glutamate induced by natural arousing stimuli and intravenous cocaine in the nucleus accumbens shell and core. J. Neurophysiol. 108, 285–299. 10.1152/jn.01167.2011 22496525 PMC3434613

[B87] Wettschureck N. Offermanns S. (2005). Mammalian G proteins and their cell type specific functions. Physiol. Rev. 85, 1159–1204. 10.1152/physrev.00003.2005 16183910

[B88] Yamagata Y. Kobayashi S. Umeda T. Inoue A. Sakagami H. Fukaya M.. (2009). Kinase-dead knock-in mouse reveals an essential role of kinase activity of Ca^2+^/calmodulin-dependent protein kinase II α in dendritic spine enlargement, long-term potentiation, and learning. J. Neurosci. 29, 7607–7618. 10.1523/JNEUROSCI.0707-09.2009 19515929 PMC6665418

[B89] Yamaguchi M. Suzuki T. Abe S. Baba A. Hori T. Okado N. (2002). Repeated cocaine administration increases GABA^B(1)^ subunit mrna in rat brain. Synapse 43, 175–180. 10.1002/syn.10037.abs 11793422

[B91] Zhang M. Han L. Xu Y. (2012). Roles of cocaine- and amphetamine-regulated transcript in the central nervous system. Clin. Exp. Pharmacol. Physiol. 39, 586–592. 10.1111/j.1440-1681.2011.05642.x 22077697

[B92] Zhen X. Goswami S. Abdali S. A. Frankfurt M. Friedman E. (2007). Estrogen-modulated frontal cortical camkii activity and behavioral supersensitization induced by prolonged cocaine treatment in female rats. Psychopharmacology (Berl) 191, 323–331. 10.1007/s00213-006-0648-0 17160679

